# Triage Management of Patients during the Outbreak of Coronavirus Disease 2019 (COVID-19)

**Published:** 2020-11

**Authors:** Reza Shirvani, Reza Heidarifar, Roghayyeh Ahangari, Monireh Mirzaie, Mahboubeh Sadat Yousefi

**Affiliations:** 1 Clinical Research Development Center, Nekouei-Hedayati-Forghani Hospital, Qom University of Medical Sciences, Qom, Iran,; 2 Department of Anesthesiology and Critical Care, Qom University of Medical Sciences, Qom, Iran.

Coronavirus disease 2019 (COVID-19), as a rapidly growing pandemic, was first reported in Iran on February 19, 2020 (1). Following the pandemic, with the spread of fear by social media, people rushed to stock up on goods, and even many rushed to hospitals and clinics. Triage of patients in emergency departments is of particular importance, as these departments have always been on the front line of care for patients. Therefore, if the triage system is not efficient, it can lead to overcrowding in hospitals (2). This overcrowding can result in the exhaustion of the medical staff, loss of patients, missing the required tests for patients, and anxiety in patients.

Overcrowding caused by erroneous triage decisions may predispose uninfected patients with contagious diseases to COVID-19 (3, 4). Accordingly, specific hospitals were initially designated to patients with COVID-19 in every city so that patients with severe conditions, such as myocardial infarction (MI), cerebrovascular accidents (CVAs), traumas, and other diseases, could be referred to other hospitals to prevent the spread of infection. Overall, the greatest challenge was the large number of patients, referred to the designated hospitals with symptoms of common cold, flu, and COVID-19.

Over the first few days of the epidemic, nearly 2000 patients visited Nekouei-Hedayati-Forghani Hospital in Qom, Iran. Therefore, patients who were referred to this hospital for cold could be exposed to COVID-19. In such cases, by developing a pre-emergency or a pre-triage system, nurses first examined the patients in a room outside the main emergency department. The nurses checked the patients’ vital signs, body temperature, and oxygen saturation and also evaluated symptoms, such as runny nose, cough, and respiratory problems. The patients were referred to general practitioners in the same ward if needed. If the symptoms were significant, they would be triaged at the main emergency department of the hospital.

In the emergency department triage, patients with acute problems were examined by specialists. After a lung CT scan and other tests, if the patient was not infected with coronavirus, he/she would be treated as an outpatient in the fast-track ward of the hospital. Patients with lymphocytopenia, thrombocytopenia, and pulmonary involvement on CT scan were referred to the inpatient wards. Overall, it seems that by accurate screening and establishing a pre-triage system before the main emergency department triage, we can significantly reduce the overcrowding of emergency wards by clients, including healthy and unhealthy ones, in times of crisis.
